# An Evaluation of a Simplified Impression Membrane Sampling Method for the Diagnosis of Microbial Keratitis

**DOI:** 10.3390/jcm10235671

**Published:** 2021-11-30

**Authors:** Tobi F. Somerville, Rose Herbert, Timothy Neal, Malcolm Horsburgh, Stephen B. Kaye

**Affiliations:** 1 Department of Eye and Vision Sciences, University of Liverpool, Liverpool L69 3BX, UK; Rose.Herbert@liverpoolft.nhs.uk (R.H.); sbkaye@liverpool.ac.uk (S.B.K.); 2 St Paul’s Eye Unit, Liverpool University Hospitals NHS Foundation Trust, Liverpool L7 8XP, UK; 3 Department of Microbiology, Liverpool University Hospitals NHS Foundation Trust, Liverpool L9 3GA, UK; Timothy.Neal@liverpoolft.nhs.uk; 4 Department of Infection Biology and Microbiomes, University of Liverpool, Liverpool L69 3BX, UK; malhorsb@liverpool.ac.uk

**Keywords:** microbial keratitis, corneal scrape, corneal impression membrane

## Abstract

The purpose of this study was to compare bacterial isolation rate using a corneal impression membrane (CIM) and a sharp instrument for obtaining corneal samples from patients with suspected microbial keratitis (MK). Data was retrospectively collected for all patients that had corneal samples taken for presumed MK between May 2014 and May 2020. Prior to May 2017 samples were collected by scraping the edges of the ulcer with a blade. From May 2017, samples were collected by placing a CIM (Millicell cell culture insert) against the ulcer. All corneal samples were processed using the same conventional diagnostic culture method. A total of 3099 corneal samples were included, of which 1214 (39.2%) were corneal scrapes and 1885 (60.9%) CIMs. Microorganisms were isolated from 235 (19.4%) and 1229 (65.2%) cases using a corneal scrape and CIM, respectively (*p* < 0.001). Of routinely described pathogenic microorganisms, there were significant increases in the isolations of *S. aureus* (2.4% to 11.3%) and *Serratia* (0.5% to 1.7%) using the CIM and no significant changes in the isolations of *S. pneumoniae* and *P. aeruginosa*. No significant differences were seen between the isolation rates of fungi or *Acanthamoeba* species. There was a significant increase in the isolation rates of other *Streptococcal* species (0.7% to 6.9%) and CNS species, specifically, *S. epidermidis* (2.1% to 26.2%), *S. capitis* (0.4% to 2.6%) *and S. warneri* (0.3% to 1.6%) using the CIM. The simplified CIM sampling method is an effective method for collecting corneal samples from patients with presumed MK in clinical practice.

## 1. Introduction

Microbial keratitis (MK) is an ophthalmological emergency that can lead to sight threatening complications such as corneal scarring, perforation, endophthalmitis and ultimately blindness [1,2,3,4]. Improving outcomes depends on accurate and rapid identification of the causative microorganism.

The need to detect bacteria, fungi, yeasts and protozoa together with the fact that there may be relatively few microorganisms in a corneal ulcer, means that an adequate sample must be obtained and cultured on a variety of different media [5]. This has led to the traditional practice of taking multiple samples by scraping the edges of the ulcer with a blade, needle or spatula and directly plating the material onto several culture media. The likely causative microorganism is often only isolated in 50% of cases using traditional scraping methods and standard conventional diagnostic culture (CDC) [6]. This, in addition to good activity of fourth generation fluoroquinolones, may explain the reluctance of some ophthalmologists to perform a corneal scrape to reach a microbiological diagnosis [7,8]. It is evident that improvements are required to both facilitate and simplify the collection of samples and the detection and diagnosis of microorganisms in suspected MK.

In 2015, we developed a minimally invasive sampling method using an impression membrane made from polytetrafluroethylene (PTFE) [9]. In 130 clinically suspected MK patients, samples were collected in a random order using a traditional corneal scraping method and a 4 mm cut-out sterile PTFE membrane that was placed onto the ulcer using sterile forceps for 5 s before being placed into enrichment culture broth and transported to the laboratory for conventional diagnostic culture. The isolation of microorganisms from cases of clinically suspected MK was found to be significantly higher using the cut-out PTFE membrane compared to corneal scrapes, 40.8% versus 26.9%, respectively [9]. We have reported that use of the PTFE membrane also improves the detection rate of HSV-1 [10] and have demonstrated that microorganisms remain stable on the PTFE membrane for prolonged storage periods [11].

In practice, however, a 4 mm punched-out PTFE membrane requires the use of fine sterile forceps to place the membrane onto the surface of the eye, meaning that specialist ophthalmic skills and a slit-lamp biomicroscope are required to undertake the sampling. The use of a 12 mm cell culture insert containing the same membrane material overcomes these practicality issues and, as such, was implemented into routine clinical practice for obtaining corneal samples in cases of suspected MK in our department in May 2017. The aim of this study was to compare the microbial isolation rate using a 12 mm PTFE Millicell cell culture insert and a sharp instrument for obtaining corneal samples from patients with suspected MK in routine practice.

## 2. Materials and Methods

Data was collected from the Department of Microbiology at the Liverpool University Hospitals NHS Foundation Trust for all patients who had corneal samples taken for clinically suspected MK between May 2014 and May 2020.

**Figure 1:**
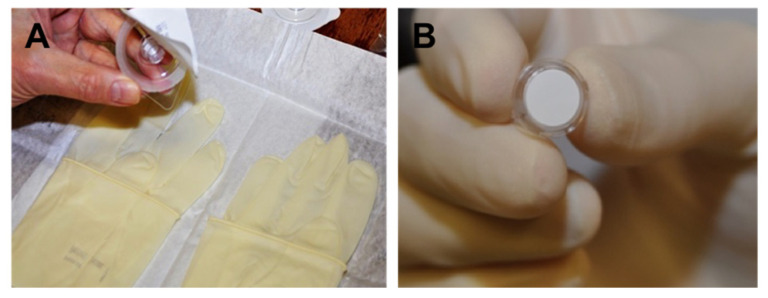
Displays the sterile pre-packaged Millicell cell culture insert being tipped onto sterile field (**A**) and the Millicell cell culture insert being held in the sampler’s dominant hand prior to sample collection (**B**).

Samples were collected from ulcers prior to May 2017 by scraping the edges of the ulcer with a number 11 blade (Swann Morton, Sheffield, UK) whilst wearing sterile gloves, as previously described [9]. The blade was placed into 0.5 mLs of enrichment culture broth, (BHI) and transported to the laboratory at room temperature. From May 2017, samples were collected from ulcers by placing the 12 mm hydrophilic PTFE membrane of a Millicell cell culture insert (CIM, corneal impression membrane) over the ulcer for 2–3 s whilst wearing sterile gloves (Figure 1). The eyelids were held apart and care was taken to avoid contact with the eyelids or conjunctiva. The CIM was placed into 9 mLs of BHI enrichment broth and transported to the laboratory. Conventional diagnostic culture was performed as previously described [9].

Microorganisms were classified as those routinely described as pathogenic in MK (*S. aureus*, *S. pneumoniae*, *Pseudomonas aeruginosa* and *Serratia*, *Candida*, *Fusarium*, *Aspergillus* and *Acanthamoeba* species) and those of unknown significance [12].

All data collected in this study were entered into an electronic database via Microsoft Excel 2018 and analysed using SPSS (version 27). The χ^2^ was used as indicated for the analysis of categorical variables. When there were fewer than five observations in any cell, Fisher’s exact test was used. Post hoc analysis was carried out using a Bonferroni correction for multiple comparisons.

## 3. Results

Over the 6-year study period, a total of 3099 corneal samples were included. The average age of patients was 49.6 years (SD 18.8). A total of 368 patients had more than one corneal sample taken during the study period, either during the same or a recurrent keratitis episode. There was a significant increase in the number of corneal samples obtained in the 3-year period following the implementation of the CIM into clinical practice compared to the 3-year period prior to its implementation, despite numbers of ophthalmic emergency attendances remaining stable (Table 1). Overall, 1214 (39.2%) of the samples were obtained using a corneal scrape and 1885 (60.9%) using a CIM, respectively (*p* < 0.001) (Table 1). No adverse events associated with the CIM were recorded during the study period.

Microorganisms were isolated from 235 (19.4%) cases using a corneal scrape and 1229 (65.2%) using a CIM (*p* < 0.001) (Table 2). Significantly higher isolation rates of both routinely described pathogenic microorganisms and microorganisms of unknown significance in MK were seen in the CIM group (Table 2). More than one isolate was identified in 30 (2.5%) and 272 (14.4%) of positive samples using a corneal scrape and CIM, respectively (Table 2).

### 3.1. Isolated Microorganisms

Of accepted MK pathogenic bacteria (12), there was a significant increase in the isolation of *S. aureus* (*p* < 0.001) and *Serratia* spp. (*p* = 0.048) using the CIM sampling method and no significant changes in the isolation of: *S. pneumoniae* and *P. aeruginosa.* No significant differences were seen between the isolation rates of *Candida, Fusarium* and *Aspergillus* species or *Acanthamoeba* species (Table 3).

Of those microorganisms of unknown clinical significance in MK, there was a significant increase in the isolation of both *Streptococcal* species other than *S. pneumoniae* and CNS species, specifically *S. epidermidis, S. capitis and S. warneri* (Table 4 and Table S1, Supplementary Data). No significant differences were seen between the isolation rates of Gram-negative bacteria and fungal species of unknown clinical significance in MK.

### 3.2. Polymicrobial Keratitis

Significantly more corneal samples in the CIM group were found to have more than one microorganism isolated, 2.4% versus 14.4%, respectively (*p* < 0.001) (Table 2). There was no significant difference in the numbers and distribution of microorganisms in each mixed sample between the corneal scrape and CIM sampling groups (Table S2, Supplementary Data). Gram-positive bacteria were the most frequent microorganism group isolated in the mixed samples in both sampling groups with CNS being the most frequently isolated organism group in all of the mixed samples. Gram-positive polybacterial infection, in which two or more species of Gram-positive bacteria were isolated, was the most common microbial growth pattern in both sampling methods. One CIM sample had mixed infection with five species of microorganism: *E. coli*, *Haemophilus haemolyticus*, *Streptococcus mitis*, an alpha-haemolytic *Streptococci* (AHS) and a *Yeast sp*. Of the mixed bacterial and fungal samples, all three corneal scrapes had mixed growth with Gram-positive bacteria, nine (3.3%) of the CIMs had mixed growth with Gram-positive bacteria and four (1.5%) had mixed growth with Gram-negative bacteria. There was only one mixed *Acanthamoeba* infection identified which had mixed growth with CNS.

## 4. Discussion

Identification of the causative microorganism is crucial for successful treatment and improving outcomes in MK. Culture of the causative microorganism remains the gold standard procedure and is necessary to determine the susceptibility profile of the pathogen. In this study, we demonstrated that the introduction of a simplified CIM sampling method into clinical practice was associated with a significant increase in the number of corneal samples collected and in microorganism isolation rates. We demonstrated the simplified CIM to be equivalent or better at isolating routinely described pathogenic microorganisms in MK compared to obtaining a corneal scrape. The inclusion of a large number of corneal samples obtained by multiple ophthalmic practitioners from a large range of patients with a wide spectrum of disease suggest the results will be applicable to corneal sampling in everyday ophthalmic clinical practice.

In the past, there have been several attempts to simplify corneal sample collection. Indirect plating methods that involve placing the sampling device into transport medium prior to plating on to agar plates reduces the need to collect multiple scrape samples and has been shown to be equivalent to directly plating corneal scrape samples in the clinic in terms of microorganism isolation rates [13,14,15]. We found that the isolation rate using the CIM (65.2%) was significantly higher than when we used a surgical blade and indirect culture, and is also higher or equivalent to those reported for other absorbent sampling devices including the direct culture of cotton tipped swabs (43.9%) [16], Mini-tip Culturettes (41.7%) [17], the indirect culture of nylon-tipped swabs (69%) [18] and the 4 mm PTFE membrane that we have previously tested (40.8%) [9]. In addition, the CIM has the benefit of being non-invasive and simple to localize to the corneal ulcer.

The spectrum of microorganisms identified in both sampling groups was similar, with Gram-positive bacteria the most frequent group isolated, and is comparable to many other studies conducted in the UK [19,20,21,22]. The CIM, in addition to demonstrating comparatively equivalent isolation rates of routinely described MK pathogenic microorganisms: *S. pneumoniae*, *P. aeruginosa*, *Candida* sp., *Aspergillus* sp. *Fusarium* sp. And *Acanthamoeba*, also demonstrated significant increases in the isolation rates of *S. aureus* and *Serratia* bacteria. This has important treatment implications as *S. aureus* is known to be the most frequently associated microorganism in recurrent MK and Gram-negative bacteria such as *Serratia* are associated with a more rapid inflammatory destructive course and worse visual outcomes [23,24,25,26].

The Biopore membrane in the cell culture insert is made from PTFE with pore size 0.4. PTFE of similar pore size was demonstrated to have the largest cellular yield when tested against other material types and the large 12 mm diameter size of the membrane in the Millicell cell culture insert increases the chances of covering the entire corneal ulcer [27]. Both of these properties could explain the increased microorganism isolation rates demonstrated in this study.

In addition to routinely described pathogenic microorganisms in MK, the CIM group demonstrated higher isolation rates of CNS and *Streptococci* species other than *S. pneumoniae* of which their pathogenic role in MK is unclear. These isolation rates were also higher than those reported using a similar one-touch method with a swab and transport medium [18]. Isolation rates of these microorganisms could be a result of several hypothesised theories. Firstly, for reasons previously discussed, the CIM sampling method is better at picking up microorganisms from the corneal ulcer itself compared to corneal scraping methods and, therefore, these microorganisms may play a larger role in the pathogenesis of MK than previously thought. The importance of identifying CNS to the species level is becoming more recognised as virulence factors such as exopolysaccharide, and lipase and protease enzymatic production are increasingly identified as potential virulence factors in MK development [28]. Secondly, the large surface area of the Millicell cell culture insert may favour the uptake of transient commensals that may be present on the patient’s lids, conjunctiva, tear film or ‘unaffected’ corneal epithelium. It is not known whether a ‘normal corneal microbiome’ exists as previous corneal sampling methodologies have been invasive and, therefore, have been able to sample unaffected and healthy corneas. Next-generation sequencing of the conjunctiva has demonstrated a diverse ocular surface microbiome made up of *Corynebacterium*, *Acinetobacter*, *Pseudomonas, Staphylococcus*, *Cutibacterium* and *Streptococcus* [29]. It is, therefore, possible that movement of the tear film across the ocular surface increases the yield of identified microorganisms during the CIM sampling process.

Polymicrobial keratitis has been previously estimated to have a 2–8% incidence rate in the literature and is associated with larger corneal infiltrate size and greater mean duration for resolution of infection [30,31]. The higher rates of polymicrobial infection seen in the CIM sampling group could similarly be a result of a better microorganism pick up rate from the ulcer itself by the CIM or represent increased inadvertent sampling of commensals as discussed. The fact that the distribution of microorganisms was not seen to differ significantly between the corneal sampling methods, however, would suggest this is not due to increased commensal organism detection.

We hypothesize that the increased numbers of corneal samples obtained in our unit post implementation of the CIM despite ophthalmic attendances remaining stable is likely to be secondary to the comparatively easier sampling methodology of the CIM for both the ophthalmic practitioner and the MK patient. The Millicell cell culture inserts are pre-sterilised and individually packed by the manufacturer making them easy to implement in a clinical setting. The cell cultures plastic mount means that the culture insert can easily be held in the hand of the user and placed over the ulcer safely without the need for sharp instruments such as forceps. Furthermore, the technique does not require the use of a slit-lamp biomicroscope, meaning that corneal samples can be obtained by allied health care professionals and in settings where access to slit-lamp biomicroscopes may be limited or not practical. The CIM requires much less patient cooperation than traditional corneal scraping methods and in the authors experience, is much better tolerated by the typical MK patient.

Although the study had a large sample size and was set over a long study duration, it is limited by its retrospective design. It is also comparing the sampling methods over two consecutive time periods and, therefore, it is unknown whether microbe detection could have been influenced by other variables that fluctuate over time such as seasonal and temperature variations [32], changing contact lens habits and hygiene [33], systemic comorbidity rates [34] and incidence and prevalence of other MK risk factors such as steroid eye drop use and ocular surface conditions [35]. UK studies looking at MK isolates over the time period used in this study are lacking. Results from five UK case series that observed MK isolates prior to this study period have conflicting results [19,20,21,22,36]. Furthermore, confounding effects of previous antimicrobial use and corneal ulcer characteristics on the isolation rates of each sampling group cannot be ruled out as the corneal scrape and CIM samples were not taken from the same corneal ulcer.

In conclusion, we demonstrated that the introduction of a simplified CIM sampling method into routine ophthalmic clinical practice was associated with a significant increase in the number of corneal samples collected and an increase in the culture positivity rate. The CIM was found to be better at isolating pathogenic microorganisms in MK and compared to traditional scraping methods, is easier to perform and requires much less patient corporation. A CIM is a safe and effective method for collecting corneal samples from patients with presumed MK in routine ophthalmic practice and is associated with higher isolation rates compared to corneal scrapes.

## Figures and Tables

**Table 1:** Number of corneal samples obtained in the 3-year periods pre- and post the implementation of corneal impression membrane (CIM) sampling into our clinical practice compared to the number of ophthalmic emergency attendances to our unit in the same time period.

	Corneal Scrapes (May 2014–April 2017)	CIM (May 2017–April 2020)
Number of corneal samples collected	1214	1885
Number of ophthalmic emergency attendances to our department	51,617	51,281
Corneal samples as a % of ophthalmic emergencies	2.4	3.7

CIM: corneal impression membrane.

**Table 2:** Isolates from cases of clinically suspected microbial keratitis over a 6-year period.

Isolated Organism	Corneal Scrapes (*n* = 1214)	CIM (*n* = 1885)	*p*-Value
Positive samples	235 (19.4%)	1229 (65.2%)	<0.001
Routinely described pathogenic microorganisms in MK	87 (7.2%)	334 (17.7%)	<0.001
Microorganisms of unknown significance in MK	152 (12.5%)	950 (50.4%)	<0.001
Mixed samples	30 (2.4%)	272 (14.4%)	<0.001

CIM: corneal impression membrane; MK: microbial keratitis.

**Table 3:** Routinely described pathogenic microorganisms identified from cases of clinically suspected microbial keratitis over a 6-year period.

Isolated Organism	Corneal Scrapes (*n* = 1214)	CIM (*n* = 1885)	*p*-Value
**Gram-positive bacteria**			
*S. aureus*	29 (2.4%)	213 (11.3%)	<0.001
*S. pneumoniae*	13 (1.1%)	33 (1.8%)	1.0
**Gram-negative bacteria**			
*P. aeruginosa*	30 (2.5%)	60 (3.2%)	1.0
*Serratia* spp.	6 (0.5%)	32 (1.7%)	0.048
**Fungi**			
*Candida* spp.	3 (0.2%)	4 (0.2%)	1.0
*Fusarium* spp.	1 (0.08%)	0	1.0
*Aspergillus* spp.	4 (0.3%)	2 (0.1%)	1.0
**Protozoa**			
*Acanthamoeba* spp.	4 (0.3%)	3 (0.2%)	1.0

CIM: corneal impression membrane.

**Table 4:** Microorganisms of unknown significance identified from cases of clinically suspected microbial keratitis over a 6-year period.

Isolated Organism	Corneal Scrapes (*n* = 1214)	CIM (*n* = 1885)	*p*-Value
**Gram-positive bacteria**	116 (9.6%)	921 (48.9%)	<0.001
Other streptococci species	9 (0.7%)	130 (6.9%)	<0.001
CNS *	76 (6.3%)	799 (42.4%)	<0.001
Others ^a^	37 (3.0%)	61 (3.2%)	1.0
**Gram-negative bacteria ^b^**	44 (3.6%)	96 (5.1%)	0.30
**Fungi ^c^**	5 (0.4%)	6 (0.3%)	1.0

CNS: Coagulase-negative *Staphylococcus*. * CNS species are shown in Table S1 in the Supplementary Data. ^a^ Other Gram-positive bacteria include: *Enterococcus* spp., *Kocuria* spp., *Micrococcus* spp., *Rothia* spp., *Aerococcus* spp., *Corynebacterium* spp., *Diphtheroid* spp., and *Bacillus* spp. ^b^ Gram-negative bacteria include: *Moraxella* spp., *E. Coli*, other unclassified *Enterobacteriaceae* spp., *Morganella* spp., *Brevundimonas* sp., *Chryseobacterium* sp., *Citrobacter* spp., *Delftia* sp., *Acinetobacter* spp., *Haemophilus* spp., *Klebsiella* spp., *Pantoea* spp., *Proteus* spp., *Raoultella* spp., *Stenotrophomonas* spp., *Achromobacter* spp. and *Alcaligenes* sp. ^c^ Fungi include: *Pencillium* spp., *Acremonium* spp., *Phoma* sp., *Scedospirium* sp., *Ulocladium* sp. and *Yeasts.*

## Data Availability

The data presented in this study are available on request from the corresponding author.

## Supplementary Materials

The following are available online at https://www.mdpi.com/article/10.3390/jcm10235671/s1: Table S1: Coagulase-negative staphylococcus isolates, Table S2: Corneal samples with mixed microorganism growth.
