# Supplementary material for: An Evaluation of a Simplified Impression Membrane Sampling Method for the Diagnosis of Microbial Keratitis
Source: J Clin Med. 2021 Nov 30;10(23):5671. doi: 10.3390/jcm10235671 (PMC8658700; doi:10.3390/jcm10235671)
Supplement: Supplementary file 1 [file jcm-10-05671-s001.zip › jcm-1486450-supplementary.pdf]

Supplementary Data

Table S1: Coagulase negative staphylococcus isolates. CIM: Corneal impression membrane.

Isolated Organism	Corneal Scrapes (n=1,214)	CIM (n=1,885)	P-value
S. epidermidis	26 (2.1%)	493 (26.2%)	<0.001
S. caprae	0	1 (0.05%)	1.0
S. capitis	5 (0.4%)	49 (2.6%)	<0.001
S. haemolyticus	0	8 (0.4%)	0.624
S. hominis	8 (0.7%)	28 (1.5%)	0.936
S. lugdunensis	1 (0.1%)	13 (0.7%)	0.182
S. pasteuri	1 (0.1%)	12 (0.6%)	0.504
S. pellenkoferi	0	2 (0.1%)	1.0
S. saprophyticus	2 (0.2%)	2 (0.1%)	1.0
S. simulans	0	2 (0.1%)	1.0
S. warneri	4 (0.3%)	31 (1.6%)	<0.001
Unclassified	31 (2.6%)	199 (10.6%)	<0.001

Supplementary Data

Table S2: Corneal samples with mixed microorganism growth. CIM: corneal impression membrane; CNS: Coagulase-negative staphylococcus. [a] Other Gram-positive bacteria includes: *Micrococcus* spp., *Rothia* spp., *Aerococcus* spp., *Lysinibacillus* spp., *Corynebacterium* spp., *Diphtheroid* spp. and *Bacillus* spp. [b] Other Gram-negative bacteria includes: *Brevundimonas* spp., *Chryseobacterium* spp., *Acinetobacter* spp., *Haemophilus* spp., *Klebsiella* spp., *Pantoea* spp., *Proteus* sp., *Raoultella* spp., *Stenotrophomonas* spp. and *Alcaligenes* sp. [c]Other fungi includes: *Ulocladium* sp. and *Yeasts.*

	Corneal Scrapes (n=30)	CIM (n=272)	P-value
Nature of infection			
2 microorganisms isolated	26 (86.7%)	241 (88.6%)	0.75
3 microorganisms isolated	4 (13.3%)	30 (11.0%)	0.71
>3 microorganisms isolated	0	1 (0.4%)	1.00
Gram positive bacteria	**29 (96.7%)**	**265 (97.4%)**	**0.81**
CNS	20 (66.7%)	196 (72.1%)	
S. aureus	7 (23.3%)	59 (21.7%)	
S. pneumoniae	3 (10.0%)	15 (5.5%)	
Other *Streptococcus* species	4 (13.3%)	101 (37.1%)	
Enterococcus spp.	1 (3.3%)	13 (4.8%)	
Others[a]	7 (23.3%)	23 (8.5%)	
Gram negative	**11 (36.7%)**	**75 (27.6%)**	**0.30**
Pseudomonas spp.	3 (10.0%)	19 (7.0%)	
Moraxella spp.	4 (13.3%)	21 (7.7%)	
E. coli	0	9 (3.3%)	
Serratia spp.	0	10 (3.7%)	
Others[b]	5 (16.7%)	22 (8.1%)	
Fungi	**3 (10.0%)**	**10 (3.7%)**	**0.13**
Candida spp.	2 (6.7%)	3 (1.1%)	
Pencillium spp.	1 (3.3%)	3 (1.1%)	
Aspergillus spp.	0	2 (0.7%)	
Others[c]	0	2 (0.7%)	
Protozoa			
Acanthamoeba spp.	**0**	**1 (0.4%)**	**1.00**
Microbial growth patterns			
Only mixed Gram-positive bacterial growth	19 (63.3%)	203 (74.6%)	0.18
Only mixed Gram-negative bacterial growth	1 (3.3%)	9 (3.3%)	1.00
Mixed Gram-negative and Gram-positive bacterial growth	10 (33.3%)	71 (26.1%)	0.40
Mixed bacterial and fungal growth	3 (10.0%)	10 (3.7%)	0.13
Protozoa and bacterial growth	0	1 (0.4%)	1.00
